# Prognostic role of the prognostic nutritional index in patients with pancreatic cancer who underwent curative resection without preoperative neoadjuvant treatment: A systematic review and meta-analysis

**DOI:** 10.3389/fsurg.2022.992641

**Published:** 2022-09-09

**Authors:** Pengcheng Zhao, Zuowei Wu, Zihe Wang, Chao Wu, Xing Huang, Bole Tian

**Affiliations:** Department of Pancreatic Surgery, West China Hospital, Sichuan University, Chengdu, China

**Keywords:** pancreatic cancer, meta-analysis, prognosis, surgery, prognostic nutritional index

## Abstract

**Background:**

The prognostic nutrition index (PNI), which has been evaluated in various kinds of cancers, offered a simple yet effective approach to predict the prognosis. The aim of this meta-analysis is to reveal the correlation between preoperative PNI and the prognosis of patients with pancreatic ductal adenocarcinoma (PDAC) who underwent curative resection.

**Methods:**

We searched the PubMed, Embase, Web of Science and Cochrane Library databases, and extracted the hazard ratio (HR) with 95% confidential interval (CI) from eligible studies. The pooled HR with 95% CI was applied to evaluate the association between PNI and overall survival (OS), recurrence-free survival (RFS).

**Results:**

A total of fourteen studies with 3,385 patients were included for meta-analysis. The results (the pooled HR: 1.664, 95% CI: 1.424–1.994, *I*² = 42.6%, *p* value **= **0.046) indicated that low preoperative PNI was closely related to poor OS. In addition, the results suggested that PNI was negatively correlated with RFS (the pooled HR: 1.369, 95%CI: 1.080–1.734). The robustness of these pooled results was verified by our subgroup analysis and sensitivity analysis. Moreover, different cutoff values among studies are responsible for the heterogeneity of pooled HR of OS through meta-regression analysis (*p* value = 0.042). Funnel plots, Begg's test (*p* value = 0.228) and Egger’s test (*p* value = 0.702) indicated no significant publication bias in OS.

**Conclusion:**

Preoperative PNI might be a promising marker to predict the prognosis of PDAC patients who underwent curative resection.

## Introduction

Pancreatic ductal adenocarcinoma (PDAC) is one of the most aggressive malignant digestive system tumors with a 5-year survival rate of approximately 9% (1). Surgical resection is taken as the only curative therapy for PDAC, and the 5-year survival rate after radical resection is about 20% (2). Despite advancements in medical technology, the prognosis of PDAC is still very poor. Therefore, it is vital to identify a marker that can predict the prognosis for patients with PDAC.

An increasing number of studies have shown that inflammation and nutrition status play a significant role in oncogenesis, progression and metastasis (3–5). Inflammatory indices, such as neutrophil-to-lymphocyte ratio (NLR) (6), platelet-to-lymphocyte ratio (PLR) (7) and controlling nutritional status (CONUT) score (8), have been applied to predict the prognosis of patients with PDAC. Prognostic nutritional index (PNI) was initially reported by Buzby and colleagues in 1980, and it calculated as 158 – 16 (ALB) – 0.78 (TSF) – 0.20 (TFN) – 5.8 (DH). (ALB is serum albumin level (g/100 ml), TSF is triceps, skinfold (mm), TFN is serum transferrin level (mg/100 ml) and DH is delayed hypersensitivity reactivity to any of three recall antigens (mumps, streptokinase-streptodornase, candida) graded as 0, 1, 2) (9). Then in 1984, Onodera T. developed a relatively simple and convenient formula of PNI to assess the risk of postoperative complications and the prognosis of gastrointestinal cancer patients after surgery, which was 10 × serum albumin (g/dl) + 0.005 × total lymphocyte count (10). Subsequently, Onodera’s PNI was widely utilized to predict the prognosis of various cancers since 2010s, including gastric cancer (11), hepatocellular cancer (12), lung cancer (13), colorectal cancer (14–16), etc. A few studies have investigated the relationship between the PNI and the prognosis of PDAC (17–19). The results of two previous meta-analysis studies indicated that low PNI was related to poorer OS. Nevertheless, they analyzed mixed patients who treated with surgery alone, chemotherapy/chemoradiotherapy alone or preoperative chemotherapy/chemoradiotherapy followed by surgery, which could bring about bias, and the conclusion might not be very reliable. These preoperative treatment regimes, especially the chemotherapy, may decline the lymphocyte count and albumin concentration *via* myelosuppression and chemotherapy toxicity, which could impact the calculation of PNI subsequently. Hence, our aim was to perform a systematic review and meta-analysis of the current published studies to evaluate the clinical significance of PNI as a preoperative prognostic factor in patient with PDAC underwent curative resection.

## Materials and methods

This systematic review and meta-analysis was conducted following the Preferred Reporting Items for Systematic Reviews and Meta-Analyses (PRISMA) statement (20).

### Search strategies

PubMed, Embase, Web of Science, and Cochrane Library databases were searched for eligible articles up to March 1st, 2022. The search was conducted using medical subject headings (MeSH) in combination with free text words. The search strategy in PubMed database was the following: (“Pancreatic Neoplasms” [MeSH Terms] OR ((“Pancreatic” [Title/Abstract] OR “pancreas” [Title/Abstract]) AND (“adenocarcinoma” [Title/Abstract] OR “carcinoma” [Title/Abstract] OR “cancer” [Title/Abstract] OR “neoplasm*” [Title/Abstract] OR “tumor” [Title/Abstract]))) AND (“Prognostic Nutritional Index” “[Title/Abstract]” OR “Prognostic Nutritional Indices” “[Title/Abstract]” OR “PNI” [Title/Abstract]).

### Inclusion and exclusion criteria

All studies included in the meta-analysis were selected according to the following inclusion criteria: (1) studies including patients who underwent curative surgical resection and confirmed as PDAC by histopathological or pathological analysis, (2) PNI was calculated using Onodera’s simplified formula, and measured before surgery, (3) studies investigating the relationship between preoperative PNI and the prognosis of PDAC, (4) hazard ratio (HR) with 95% confidence interval (CI) or other necessary data was available, and (5) studies written in English and published in full-text. The exclusion criteria were as follows: (1) patients received any preoperative neoadjuvant chemotherapy, chemoradiotherapy, or immunotherapy, (2) abstracts, case reports, editorials, letters, systematic reviews, and comments, (3) studies with incomplete data, (4) studies enrolled the overlapped or same population, and (5) duplicate studies.

### Data extraction

Two investigators (PCZ and ZWW) independently extracted necessary data from included studies and any disagreements were resolved by discussion till reach consensus. The following data were extracted from each study: first author, publication year, country, study design, age of the study population, male/female, sample size, cutoff value of PNI, tumor stage, duration of follow-up, operation, outcome measures, type of analysis, and recurrence-free survival (RFS) and overall survival (OS) with HR and their 95% CI. Because of confounding factor adjustment, the multivariate analysis was preferred when the HRs for OS or RFS were obtained using both univariate and multivariate analyses. If HR with 95% CI was not provided in original studies, we extracted from the survival curve by using Engauge Digitizer software (https://markummitchell.github.io/engauge-digitizer/).

### Quality assessment

The Newcastle-Ottawa quality assessment Scale (NOS) was used to evaluate the quality of included studies. The NOS consists of 3 aspects: selection (4 points maximum), comparability (2 points maximum) and outcomes (3 points maximum). Studies with a score of six or higher were considered as high-quality studies (21). This work was also performed independently by our two investigators (PCZ and ZWW). (Supplementary Table S1)

### Statistical analysis

Meta-analysis was conducted using Stata 14.0 software (https://www.stata.com/stata14/). The pooled HR with 95% CI was used to evaluate the relationship between the preoperative PNI and the outcome in patients with PDAC. The heterogeneity of pooled HR was accessed using Cochran’s *Q* test and Higgins *I*² statistic. *Q* test *p* value < 0.1 or *I*² > 50% was considered significant heterogeneity and random-effect model was applied to estimate the pooled HR. While heterogeneity was not significant (*Q* test *p* value > 0.1 or *I*² < 50%), a fixed-effect model was used. To reduce and explain the heterogeneity of OS among studies, subgroup analyses, meta-regression analysis and sensitivity analysis were applied. Furthermore, publication bias was visually checked through funnel plot, and then quantitatively analyzed by Begg’s and Egger’s tests. All statistical tests were two-sided, and *p* value less than 0.05 were defined as statistically significant.

## Results

### Study selection

**Figure 1:**
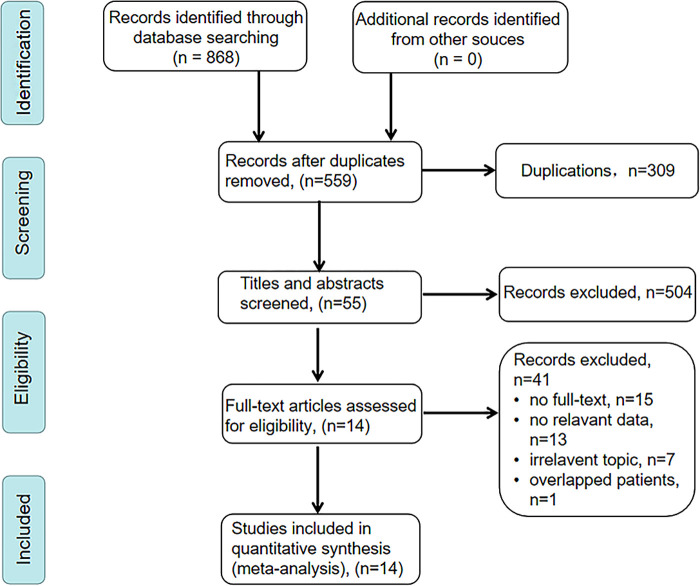
PRISMA flow diagram of eligible studies selection.

We searched PubMed, Embase, Web of Science, and Cochrane Library databases, and a total of 868 articles were initially retrieved. After removing 309 duplicates, 559 articles remained. After screening the titles and abstracts, 455 articles were excluded for being irrelevant topics, reviews or meta-analysis, conference abstracts, or meeting. Among the remained 104 articles, only 55 articles were performed among patients who underwent curative resection. Finally, 14 articles met our inclusion criteria and 3,385 patients were included in this meta-analysis (17–19, 22–31). The detailed selection process was illustrated in Figure 1.

### Clinical characteristic of enrolled studies

The main characteristics of included studies were presented in Table 1. These included studies were retrospective studies, and mainly published in the past ten years. All included studies used the Onodera’s PNI. Twelve of fourteen studies were from Japan, and two studies were from China. The sample size of enrolled studies varied from 46 to 589. In all selected articles, the correlation between PNI and OS was presented, while RFS was additionally analyzed in two studies. The preoperative PNI cut off value were not consistent ranged from 36 to 53.10. Multivariate analyses were conducted in ten of fourteenth studies. The scores of study quality assessed by NOS ranged from 6 to 8.

**Table 1:** The main characteristics of included studies.

Year	Author	Country	Study type	Sample size (low PNI/high PNI)	Tumor stage	Operation (PD/DP/MP/TP)	Median follow-up (months)	Cutoff value	Postoperative chemotherapy, *n*=	Analysis model	Outcome	NOS
2011	Kanda M.	Japan	R	74/194	I–IV	195/48/0/25	NA	45	NA	M	OS	6
2016	Asaoka T.	Japan	R	21/25	I–III	Only PD	NA	47	26	M	OS	6
2016	Watanabe J.	Japan	R	9/37	I–III	Only PD	NA	40	30	U	OS	6
2018	Abe T.	Japan	R	206/123	I–III	214/96/0/19	NA	45	286	M	OS	7
2019	Ikeguchi M.	Japan	R	24/26	I–III	33/15/0/2	NA	46	NA	M	OS	6
2019	Ikuta S.	Japan	R	90/46	I–IV	83/53/0/0	16.8	48.8	112	U	OS	8
2019	Onoe S.	Japan	R	18/147	I–III	Only PD	59.6	38	66	M	OS	7
2020	Hoshimoto S.	Japan	R	92/119	I–IV	119/80/0/12	19.0	47.25	113	U	OS	7
2020	Mao Y. S.	China	R	75/231	I–III	NA	NA	45	NA	M	OS	6
2020	Xu S. S.	China	R	333/249	I–III	243/339/0/0	NA	53.10	477	U	OS	7
2021	Abe T.	Japan	R	26/133	I–III	69/79/0/11	27.6	40	105	M	OS/RFS	7
2021	Itoh S.	Japan	R	256/333	I–III	394/179/0/16	NA	46	NA	M	OS/RFS	6
2021	Onoe S.	Japan	R	62/125	I–IV	125/40/0/22	39.8	36	147	M	OS	7
2021	Terasaki F.	Japan	R	122/185	I–IV	237/70/0/0	NA	50	219	M	OS	6

R, retrospective; PD, pancreaticoduodenectomy; DP, distal pancreatectomy; MP, medial pancreatectomy; TP, total pancreatectomy; NA, not applicable; M, multivariate; U, univariate; OS, overall survival; RFS, recurrence-free survival; NOS, Newcastle–Ottawa scale.

### Relationship between PNI and OS

**Figure 2:**
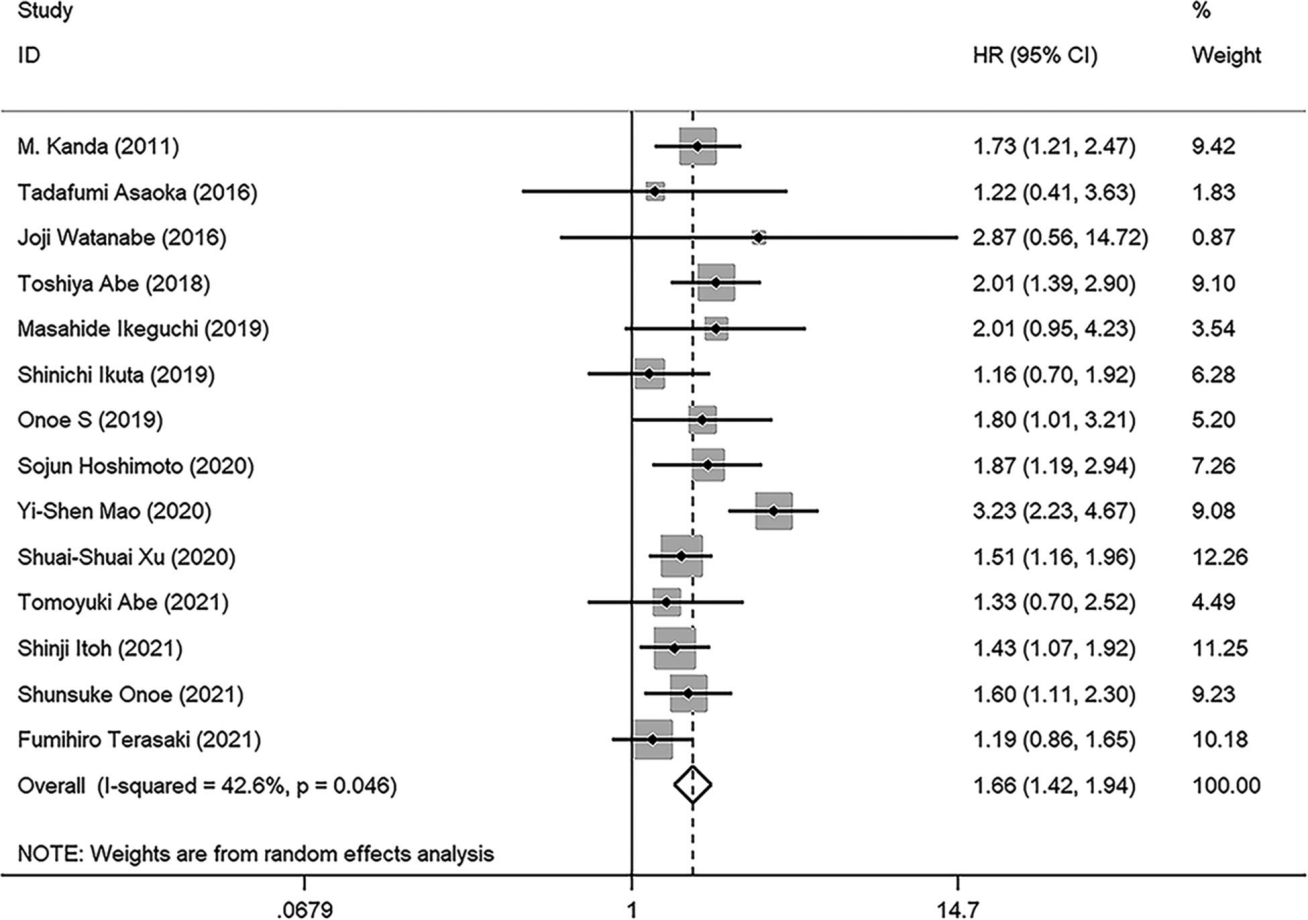
Forest plot of the association between PNI and OS. (OS, overall survival; HR, hazard ratio; CI, confidence interval).

As illustrated in Figure 2, a total of 14 studies were enrolled in this meta-analysis, and the results indicated that patients with low PNI had significantly worse OS (HR = 1.664, 95%CI: 1.424–1.944, *I*^2 ^= 42.6%, *p* value =** **0.046). Subgroup analyses were conducted based on the sample size, tumor stage, cutoff value, and analysis model, and the results revealed that low PNI was still associated with inferior OS in all subgroups. Meta-regression analysis was performed to explore the heterogeneity, and the *p* value of cutoff value subgroup was 0.042, which indicated that using different cutoff value among studies might be the source of heterogeneity. Meanwhile, PNI was confirmed as an independent preoperative prognostic factor of OS in 6 studies (17–19, 23, 26, 28). All results of subgroup analyses and meta-regression analyses were shown in Table 2.

**Table 2:** The results of subgroup analyses and meta-regression analyses.

Subgroup	References	Patients, *n*=	Random-effects model		Fixed-effects model		*p* value (heterogeneity)	*I*^2^, %	*p* value (meta-regression)
			HR, 95%CI	*p* value	HR, 95%CI	*p* value			
Sample size									
≤200	(23, 24, 28–32)	793	1.524 (1.218–1.908)	<0.001	1.524 (1.218–1.908)	<0.001	0.814	0	0.502
>200	(17–19, 22, 25–27)	2592	1.742 (1.386–2.190)	<0.001	1.685 (1.486–1.911)	<0.001	0.004	68.6	
Tumor stage									
I–III	(17, 18, 24–26, 28, 30–32)	2276	1.812 (1.444–2.273)	<0.001	1.767 (1.533–2.037)	<0.001	0.047	49	0.257
I–IV	(19, 22, 23, 27, 29)	1109	1.482 (1.231–1.784)	<0.001	1.480 (1.245–1.759)	<0.001	0.336	12.2	
Cutoff value									
≤45	(18, 19, 23, 24, 26, 28, 31)	1464	1.952 (1.546–2.456)	<0.001	1.974 (1.670–2.333)	<0.001	0.111	42.0	0.042
>45	(17, 22, 25, 27, 29, 30, 32)	1921	1.432 (1.238–1.657)	<0.001	1.432 (1.238–1.657)	<0.001	0.638	0	
Analysis type									
Multivariate	(17–19, 22–24, 26, 28, 30, 32)	2410	1.714 (1.398–2.101)	<0.001	1.696 (1.489–1.932)	<0.001	0.022	53.6	0.587
Univariate	(25, 27, 29, 31)	975	1.525 (1.244–1.871)	<0.001	1.525 (1.244–1.871)	<0.001	0.472	0	

### Relationship between PNI and RFS

**Figure 3:**
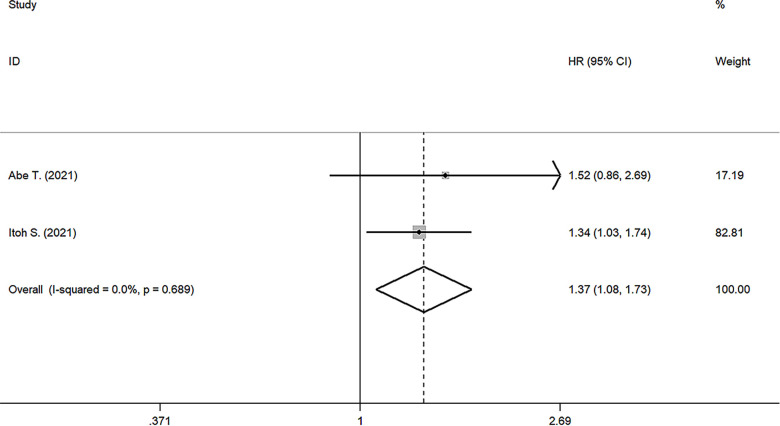
Forest plot of the association between PNI and RFS. (RFS, recurrence-free survival; HR, hazard ratio; CI, confidence interval).

Two studies reported the prognostic value of PNI for RFS (17, 24), the pooled results were: HR: 1.369, 95%CI: 1.080–1.734, *I*^2 ^= 0, *p* value = 0.689, which suggested patients with lower PNI had shorter RFS than those with high PNI (Figure 3).

### Sensitivity analysis and publication bias

**Figure 4:**
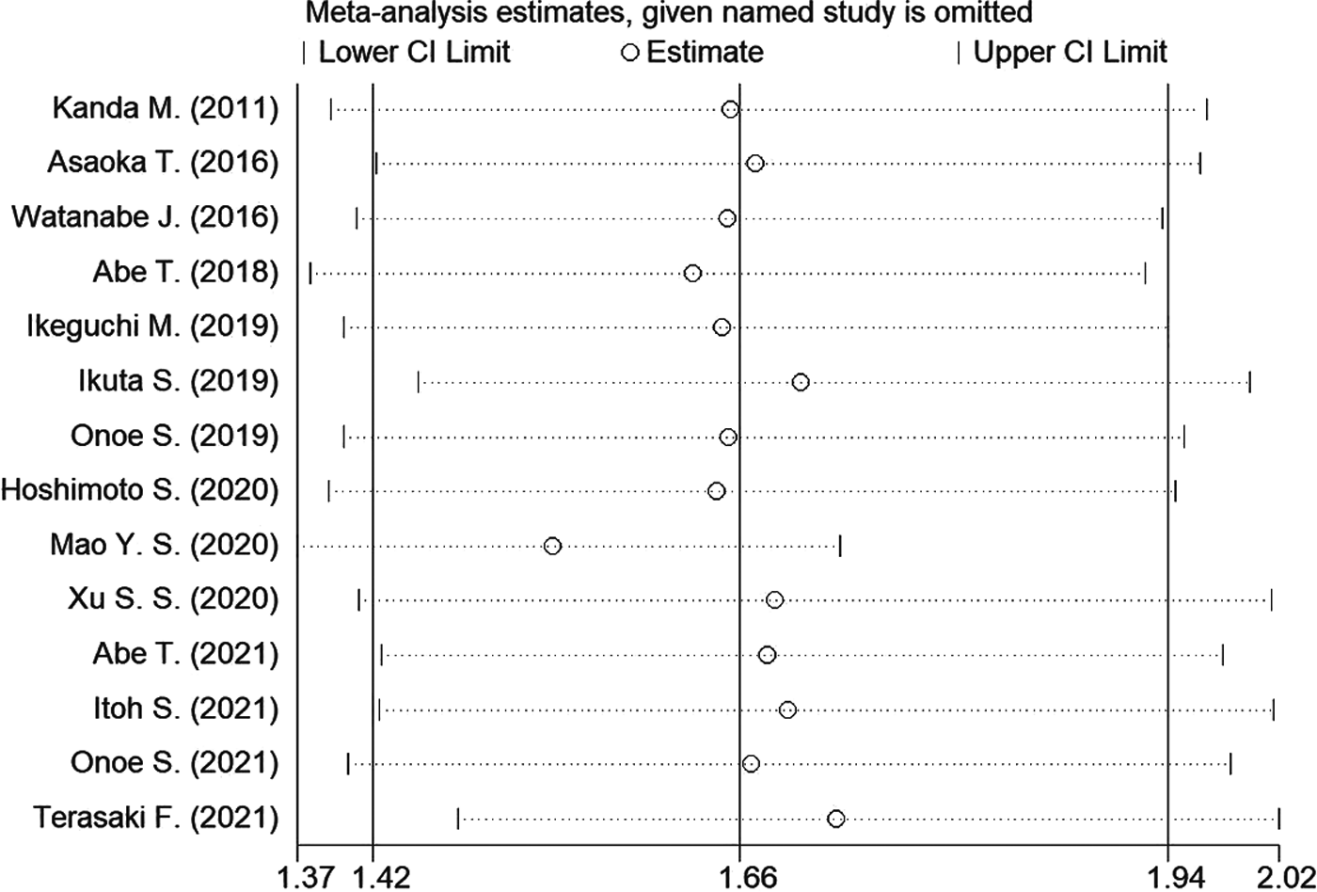
Sensitivity analysis of the relationship between PNI and OS. (OS, overall survival).

**Figure 5:**
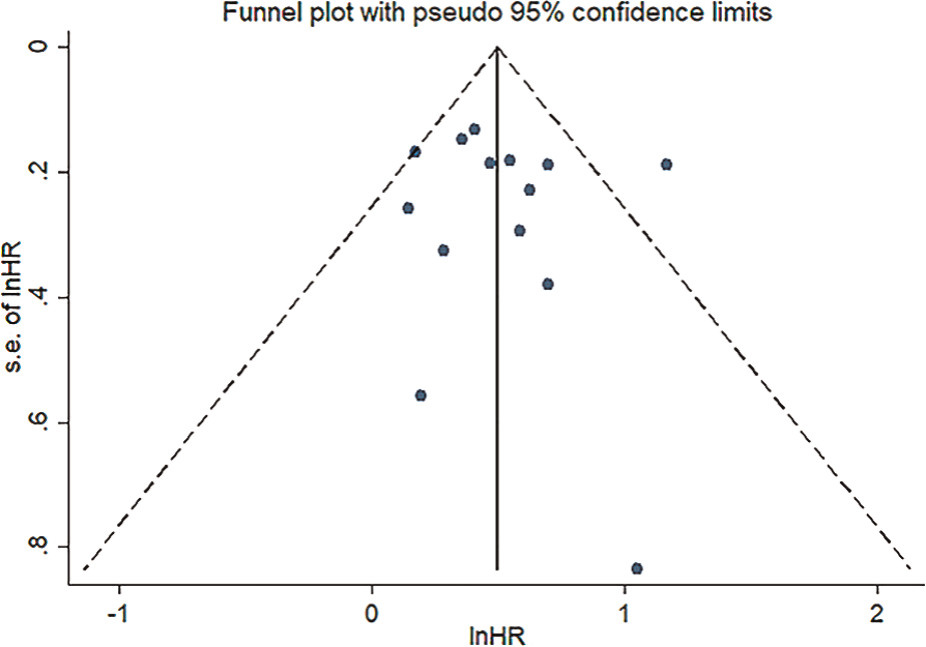
Funnel plot illustrating publication bias test result.

Sensitivity analysis was conducted to assess the effect of individual studies on the pooled HR of OS, and the result revealed that omitting any individual studies had no significant effect on the pooled HR (Figure 4). Furthermore, publication bias was investigated, and there was no obvious asymmetry in the funnel plot upon visual inspection (Figure 5), then Begg's and Egger's tests yielded *p* values of 0.228 and 0.702, respectively, which indicated that there was no distinct publication bias among included studies.

## Discussion

Prognostic nutritional index (PNI) was previously known as a nutritional evaluation index, it has recently been reported to be useful to estimate postoperative morbidity and predict the prognosis in PDAC patients. In our meta-analysis, fourteen studies with a total of 3,385 patients were included (17–19, 22–32). The pooled results showed that lower preoperative PNI was association with poorer OS and RFS. Moreover, results from subgroup analyses and sensitivity analysis further validated the robustness of pooled results.

According to the meta-regression analysis, the diversity of cutoff value might be the source of heterogeneity. There were several methods to determine cutoff value. Among these studies, five studies were defined with a receiver operating characteristic curve (ROC) (17, 24, 27, 29, 31), three with the minimum *p* value approach (22, 25, 28), and one set the worst tertile of PNI as cutoff value (23). In other studies, mean or median value was used in two studies (30, 32), and three had no clear explanation (18, 19, 26). As a consequence, the cutoff value for PNI ranged from 36 to 53.10. The ROC curve approach maybe the most common way to identify cutoff value. The ROC could reflect the 1-specificity values (false positive rate, *X*-axis) and the sensitivity values (true positive rate, *Y*-axis) for each potential threshold, and we were able to determine the cutoff value with high accuracy. The minimum *p* value approach, also called maximal Chi square statistics approach, was another common method. Each value was assessed as potential threshold, and Chi squared tests were utilized. The maximal Chi square value corresponding threshold was recognized as the optimal cutoff value, however, the type I error rate might be higher due to multiple testing of this method (33, 34). The best way to determine cutoff value is still up for debate, and we have not been able to come to a consistent conclusion. Hence, more multi-institutional data analyses were required to reach a definitive conclusion about cutoff values.

PNI was calculated by albumin and lymphocyte. Albumin, mainly synthesized by hepatocytes, was closely related to nutritional status. Hypoalbuminemia showed the level of malnutrition and cachexia of cancers patients. Some cytokines in tumor microenvironment, such as TNF-α, played an optimal role in the pathogenesis of malnutrition in pancreatic cancer (35). TNF-α could selectively inhibit the gene expression of albumin, causing hypoalbuminemia. Some research indicates that nutrition is a crucial determinant of immune response, which may be impaired by hypoalbuminemia (36). Thus, low levels of serum albumin can be recognized as a marker of poor prognosis in PDAC (37, 38). It was widely acknowledged that lymphocytes were indispensable components of immune system and tumor microenvironment (4). Immune surveillance was considered as the vital part of anti-tumor immunity, however, tumor cells might escape the surveillance by reducing CD4+ and CD8+ lymphocytes causing lymphocytopenia (39). Low lymphocyte counts lead to insufficient immunological responses in tumor microenvironment and result in cancer progression. In addition, the impairment of lymphocyte subsets may be reversed when resecting primary tumor (40). What is more, malnutrition and weak immune could increase the risk of postoperative complications, such as bleeding, pancreatic fistula and infection (26, 41). Therefore, PNI might be a promising predictor of prognosis in patients with PDAC.

There were several limitations in our meta-analysis. Firstly, all studies we selected are retrospective in design, so the potential bias was not inevitable. Secondly, the ethnicity of all included patients is Asian, and we expect the more similar studies can be conducted in Caucasians and Africans. Thirdly, HR and 95% CI of one study was estimated according to the survival curve (31), which might not be very accurate. It would affect the pooled HR. Fourthly, patients with tumor located in pancreas head usually underwent pancreaticoduodenectomy, while distal pancreatectomy or medial pancreatectomy were always performed when tumor locates in pancreas body or tail. Different surgical procedures were associated with different prognosis, and it may result in bias. Finally, all include studies were published in English, and potential publication bias cannot be ignored.

## Conclusion

To sum up, our meta-analysis revealed that PDAC patients with lower preoperative PNI level had a worse prognosis. The limitation of this study also cannot be overlooked, and more well-designed studies with large sample size and different ethnicity are required to overcome these limitations.

## Data Availability

The original contributions presented in the study are included in the article/**Supplementary Material**, further inquiries can be directed to the corresponding author/s.
