# Supplementary material for: Prognostic role of the prognostic nutritional index in patients with pancreatic cancer who underwent curative resection without preoperative neoadjuvant treatment: A systematic review and meta-analysis
Source: Front Surg. 2022 Sep 9;9:992641. doi: 10.3389/fsurg.2022.992641 (PMC9500291; doi:10.3389/fsurg.2022.992641)
Supplement: Supplementary file 1 [file Table_S1.doc]

**Supplementary information**

**Table S1. The Newcastle-Ottawa scale for quality assessment of include studies.**

| **Study** | **Selection** | | | | **Comparability** | **Outcome** | | | **Total score** |
| --- | --- | --- | --- | --- | --- | --- | --- | --- | --- |
|  | Representative-ness  of the exposed  cohort | Selection  of the  non-exposed  cohort | Ascertainment  of exposure | Demonstration  that outcome  of interest was  not present at  start of study | Comparability  of cohorts on  the basis of  the design or  analysis | Assessment  of outcome | Was follow-  up long  enough for  outcomes to occur | Adequacy  of follow  up of  cohorts |  |
| **Total score** | 1 | 1 | 1 | 1 | 2 | 1 | 1 | 1 | 9 |
| Kanda M. | 1 | 1 | 1 | 1 | 2 | 1 | 0 | 0 | 7 |
| Asaoka T. | 1 | 1 | 1 | 1 | 0 | 1 | 1 | 0 | 6 |
| Watanabe J. | 1 | 1 | 1 | 1 | 1 | 1 | 1 | 0 | 7 |
| Abe T. | 1 | 1 | 1 | 1 | 1 | 1 | 1 | 0 | 7 |
| Ikeguchi M. | 1 | 1 | 1 | 1 | 0 | 1 | 1 | 0 | 6 |
| Ikuta S. | 1 | 1 | 1 | 1 | 1 | 1 | 1 | 1 | 8 |
| Onoe S. | 1 | 1 | 1 | 1 | 0 | 1 | 1 | 1 | 7 |
| Hoshimoto S. | 1 | 1 | 1 | 1 | 0 | 1 | 1 | 1 | 7 |
| Mao Y. S. | 1 | 1 | 1 | 1 | 0 | 1 | 1 | 0 | 6 |
| Xu S. S. | 1 | 1 | 1 | 1 | 1 | 1 | 1 | 0 | 7 |
| Abe T. | 1 | 1 | 1 | 1 | 1 | 1 | 1 | 0 | 7 |
| Itoh S. | 1 | 1 | 1 | 1 | 0 | 1 | 1 | 0 | 6 |
| Onoe S. | 1 | 1 | 1 | 1 | 0 | 1 | 1 | 1 | 7 |
| Terasaki F. | 1 | 1 | 1 | 1 | 0 | 1 | 1 | 0 | 6 |
